# Active and break spells of summer monsoon over Bangladesh

**DOI:** 10.1016/j.heliyon.2023.e20347

**Published:** 2023-09-20

**Authors:** Zannatul Ferdoushi, D.A. Quadir, S.M. Quamrul Hassan

**Affiliations:** a Department at Meteorology, University of Dhaka, Dhaka, 1000, Bangladesh; b NPI University Bangladesh, Manikganj, 1800, Bangladesh; c Bangladesh Meteorological Department, Agargaon, 442101, Dhaka, Bangladesh

**Keywords:** Summer monsoon, Active and break spells, WRF model, Synoptic condition

## Abstract

This study examines the intraseasonal variability of the southwest summer monsoon over Bangladesh using rainfall data from rain gauge stations of the Bangladesh Meteorological Department (BMD) collected over 30 years (1988–2017). In this paper, active and break spells are deﬁned as periods during the peak monsoon months of July and August, in which the daily precipitation lasts for three or more days at 0.5 above and below the daily climatological cycle. The active and break phases of the monsoon over a period of 10 years (2008–2017) were also analyzed by The Weather Research and Forecasting (WRF) model simulation for analysing synoptic conditions. The model simulations for each year were done for the period of 1 May to 30 September with 1 May as the initial condition with a single domain of 30 km resolution and 19 vertical levels. The ﬁnal operational global analysis data from the Global Forecasting System of National Centers for Environment Prediction (NCEP-FNL) with resolution 1° × 1° is used for model simulation. The model-simulated daily rainfall, Sea Level Pressure (SLP), wind pattern at 850 hPa, 200 hPa, and Outgoing Longwave Radiation (OLR) are compared with the observations from Tropical Rainfall Measuring Mission (TRMM), ERA5 (ERA5 is the European Centre for Medium-Range Weather Forecasts fifth-gen global atmospheric reanalysis data), and Kalpana-1. This study also finds that the increase in rainfall is concurrent with a southwesterly wind and the decrease of rainfall simultaneously occurs with a southeasterly wind. Active days were found to have lower OLR values and lower SLP than break days.

## 1 Introduction

The Southwest Summer Monsoon is a component of both the South Asian Summer Monsoon [1,2] and the global circulation system, which significantly influences the climate of the Indian subcontinent [3]. Active and break phases, referred to as active phases and dry stretches alternately, are distinctive features of the southwest summer monsoon [4]. The latitudinal oscillations of the monsoon trough result in oscillations between active and break periods during the monsoon season [2].

Bangladesh is situated in the tropical monsoon region and stretches from 20°45′N to 26°40′N and from 88°05′E to 92°40′E [5]. This country is crossed by 230 rivers, of which 57 originate outside its borders, with the majority flowing from north to south [6]. Bangladesh has involvement with two different environments, one in the Bay of Bengal in the south and another in the Himalayas in the north [7] which acts like a barrier and enhances the rainfall amount in the northeastern part of this country. Due to its geographic location, Bangladesh experiences the most yearly rainfall among the South Asian Association for Regional Cooperation (SAARC) nations as well as the most country-average monsoon rainfall [8]. The monsoon season accounts for about 70–80% of all of this country's annual precipitation [9]. External surface boundary forcing and internal dynamics influence monsoon rainfall variability [10]. According to the Intergovernmental Panel on Climate Change, Bangladesh's precipitation may increase by five to six percent by the year 2030 [11]. Failure or success of monsoon determines agricultural production in the country [12]. In a large-scale environment, the active and break regimes significantly influence Bangladesh's flood and drought conditions. One of the summer monsoon's weather systems is semi-permanent, and the other is permanent [13]. It has a strong association with El Nino Southern Oscillation (ENSO) events through oceans-atmospheric interaction [14].

Active spells and break spells of the southwest summer monsoon have been extensively studied **[e.g.** [[15], [16], [17], [18]]. However, only a limited number of works have been found concerning active and break spells of the southwest summer monsoon in Bangladesh. Investigation about the arrival and withdrawal dates of the summer monsoon in Bangladesh found the monsoon season onsets in mid-June and withdrawal in mid-October [5]. Blanford first noted the variation in rainfall over the monsoon trough zone between periods "at the height of showers" and "intervals of dryness" [15]**.**

Active and break phases are defined as times during the peak monsoon months of July and August when the normalized anomaly of the rainfall over a crucial region known as the monsoon core zone exceeds 1 or is less than 1, respectively, if the requirement is met for at least three days in a row [16]**.** Following the same criteria active and break periods within the monsoon core region were determined for the years 1901–2014 [17]. When daily rainfall over central India (CIR, 73°-78°E, 20°-25°N) is greater (lesser) than 0.5 standard deviations from the mean of July–August each year for a minimum of three consecutive days is mentioned active (break) [2]. To identify breaks, researchers applied criteria of 2.5 and 7.5 mm/day over the western and eastern zones, respectively, in the monsoon zone for the period 1901–1989 [18]. Breaks were identified based on the 850 hPa wind speed at a single grid point at 15°N, 90°E [19]. Breaks were classified as days with positive OLR anomalies over central and northwest India (73°-82°E, 18°-28°N) that exceeded 10 W/m^2^ [20].

Summer monsoon rainfall data for Central India (21–27°N, 72–85°E) were analyzed for the longest instrumental period from 1951 to 2003 to identify active and break monsoon periods [[20], [21], [22]]. An analysis of decadal mean rainfall indicated that the decades 1961–1970 and 1981–1990 were wet, while the decades 1971–1980, 1991–2000, and 2001–2010 were dry [7]. The active/break cycle of rainfall fluctuation in Bangladesh during the 1995 summer monsoon season resulted from the northward propagation of the monsoon trough [23]. Seasonal monsoon rainfall is composed of two prominent intraseasonal oscillations with periods of 45 and 20 days [24].

Using several models, the diurnal fluctuations of precipitation and cloudiness during the active and break phases of the summer monsoon in 2000 were investigated [25]. Using the WRF-ARW (Advanced Research WRF) model, found rainfall was positively correlated with Indian Meteorological Department (IMD) rainfall at the onset of the Indian Summer Monsoon [25]. Contrasting large-scale atmospheric features during active and break periods were observed in the WRF model control run, demonstrating its capability to forecast active (break) phases [[26], [27]]. The WRF-ARW model, with a resolution of 30 km, successfully simulated the primary characteristics and fluctuations of the Indian Summer Monsoon in ENSO phases [28]. The Mix99 CPSs scheme for the RegCM (Regional Climate Model) model performs well in simulating the synoptic features during the phases of monsoon from 1986 to 2010 [29]. It is noteworthy that the model could simulate some regional-scale features, including a heat low, ridge lines, trough lines, well-defined low-level cyclonic circulation, upper troposphere anticyclonic circulation, subtropical westerly jet, and orographic precipitation [28]. The diurnal variation of monsoon rainfall across the Indian region was characterized incorrectly by WRF model simulations but produced positive findings when they were used to simulate intraseasonal rainfall fluctuations over the central Indian region [30]. The focus of this study is to identify active and break phases of the summer monsoon (1988–2017) over Bangladesh and to investigate the associated weather scenarios (2008–2017) using the WRF-ARW model.

## 2 Data used, model Experimental set up and methodology

### 2.1 Data

#### 2.1.1 Bangladesh meteorological department (BMD) station data

**Fig. 1:**
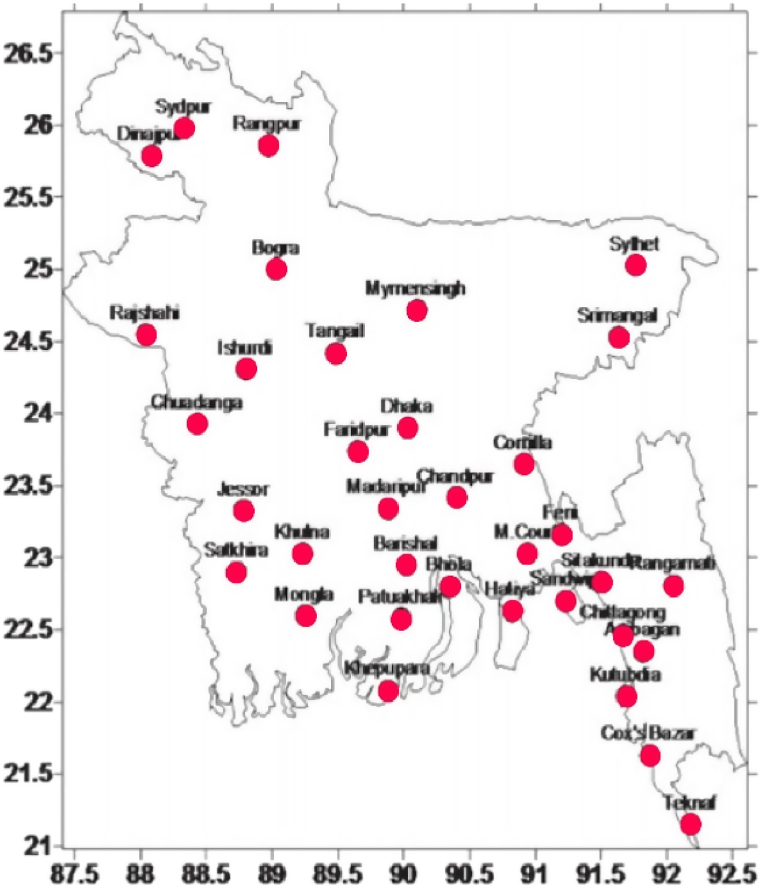
The locations of BMD's 35 rain gauge stations [31].

The observed daily rainfall data of 35 rain gauge stations of Bangladesh during the period 1988–2017(30 years) were collected from the Climate Division of BMD. This dataset was subjected to a visual inspection but some data are suspected; those data were filled up by inverse square distance weighted interpolation technique. The location of rain gauges over Bangladesh is shown in Fig. 1.

Data link: http://bmd.wowspace.org/

#### 2.1.2 NCEP FNL data

NCEP‐FNL is the ﬁnal operational global analysis data from the Global Forecasting System of the National Centers for Environment Prediction. NCEP-FNL (Final) is a product of the Global Data Assimilation System (GDAS). GDAS continuously collects observational data from the Global Telecommunications System (GTS), and other sources, for many analyses. NCEP-FNL (Final) data resolution on 1° × 1° degree prepared operationally every 6 h [30]. This 1° × 1° data (2008–2017) is used for this study in which the data format is in gridded binary (grib2).

Data link: https://rda.ucar.edu/

#### 2.1.3 TRMM data

Tropical Rainfall Measuring Mission (TRMM), daily precipitation (TRMM_3B42_daily_v7) 0.25° × 0.25° resolution data is available over the study area. This data provides total daily precipitation. TRMM Multi-Satellite Precipitation Analysis is applied as an algorithm in the 3B42_daily 7 versions. The dataset currently contains two products, the first one is three-hourly combined microwave-IR estimates with gauge adjustment and the second one is monthly combined microwave-IR-gauge estimates of precipitation computed on quasi-global grids [32]. Model simulated rainfall is compared with TRMM rainfall.

Data link: https://giovanni.gsfc.nasa.gov/giovanni/

#### 2.1.4 ERA5 data

The European Centre for Medium-Range Weather Forecasts (ECMWF) provides ERA5 data which is the fifth generation global atmospheric reanalysis data. ERA5 1 h's interval data (25° × 0.25°) [33] is used to compare the model simulated wind and sea level pressure during 2008–2017.

Data link: https://cds.climate.copernicus.eu/

#### 2.1.5 IITM Kalpana-1(K-1) OLR data

Outgoing Longwave Radiations (OLR) are estimated by utilizing infrared (10.5–12.5 μm) and water vapor (5.7–7.1 μm) radiances of Very High-Resolution Radiometer (VHRR) instrument onboard Kalpana-1 satellite stationed at 74° E. The VHRR images were obtained from the archival of the National Satellite Data Centre of the India Meteorological Department, New Delhi. Outgoing Longwave Radiations (OLR) data are available at 3-h intervals starting May 2004 over the Indian region (400S - 400 N, 250E - 1250E) in a regular latitude-longitude grid of resolution 0.25 × 0.25° [34].

Data link: https://www.tropmet.res.in/DataArchival-51-Page.

### 2.2 Methodology

#### 2.2.1 Statistical analysis

Daily rainfall data are available for 35 rain gauge stations of the BMD. For the identification of active and break spells, firstly, the station-wise daily average rainfall was calculated continuously from June to September from 1988 to 2017. The normal value of rainfall (mm) per day was collected from BMD. Then the standardized rainfall anomaly was calculated for a particular year and then divided by the standard deviation for a particular year. The paper ″Intraseasonal variation of monsoon activities associated with the rainfall over Bangladesh during the 1995 summer monsoon season by Oshawa et al. (2000)″ is used as a reference for the threshold value + 0.5 to −0.5 of standardized rainfall over Bangladesh to identify the active and break days.

#### 2.2.2 WRF model configuration for this study

**Fig. 2:**
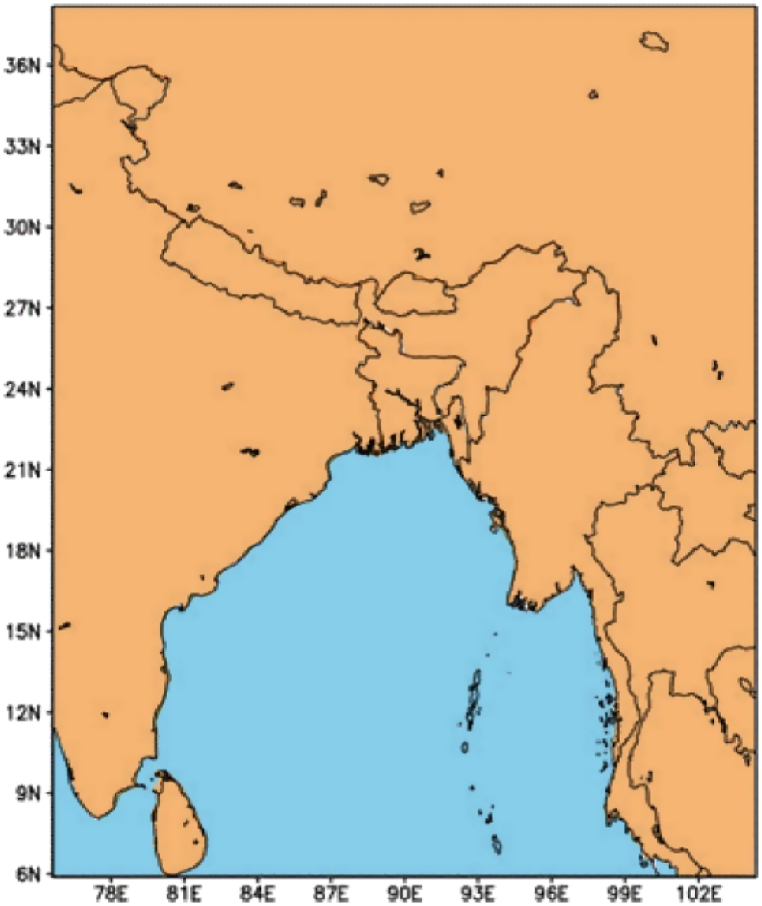
Domain for the WRF model simulation.

The simulations in this study are conducted with the WRF model, version 4.0. For this study, the model was configured with 30 km horizontal resolution and WRF Single-Moment 3-class scheme as a microphysics scheme which is suitable for mesoscale grid sizes. The model was simulated with 19 vertical levels keeping the top of the model at 100 hPa. The Kian-Fritsch scheme is used as a Cumulus Parameterization, Rapid Radiate Transfer Model (RRTM) scheme, and the Dudhia scheme are used as longwave and shortwave radiation schemes respectively. For the surface layer and planetary boundary layer, Unified Noah Land Surface Model (LSMs) and Yonsei University (YSU) schemes are used respectively. The model domain extends between 75°E to 105°E zonally and 6°S to 39°N meridionally. NCEP-FNL data having a resolution of 1° × 1° is used for model simulation. The model has been integrated starting from May to September for every year from 2008 to 2017. The model simulation corresponding to May to September is used in the present study allowing 1 month (May) as a spin-up time. For the dynamical equilibrium between the lateral forcing and the internal physical dynamics of this model simulation, a one-month spin-up time is enough [35]. The model output is taken at a daily interval corresponding to 00 UTC. The Southwest monsoon extends across a significant expanse, and for the purposes of this study, we utilize the WRF model simulation domain illustrated in Fig. 2.

## 3 Result and discussion

### 3.1 Active and break spells based on rainfall

The peak monsoon months are July and August, during which the standardized anomaly of rainfall over Bangladesh either exceeds 0.5 or falls below −0.5, respectively. This condition must persist for a minimum of three consecutive days, which are referred to as the active and break phases. To identify these active and break phases, this study uses observational rainfall data collected from BMD from 1988 to 2017. Typically, active and break monsoons occur due to the movement of the Intertropical Convergence Zone (ITCZ). The identified active and break phases of the summer monsoon are listed in Table 1.

**Table 1:** Active and break phases of monsoon over Bangladesh from 1988 to 2017 (J and A represent July and August respectively).

Year	Active phases	Break phases
1988	6J–9J, 9A–11A	13J–22J, 27J-2A
1989	1J–4J, 28J–30J	9J–11J, 16J–27J, 3A–6A, 12A–15A, 23A–29A
1990	4J–6J, 27J–29J	16J–19J, 22J–25J, 21A–25A, 28A–31A
1991	18J–20J	9J–16J, 24J–29J, 11A–15A
1992	10J–12J	1J–3J, 13J–15J, 23J–27J, 2A–5A,22A–25A, 29A–31A
1993	20J–24J, 20A–24A	3J–5J, 11J–14J, 25J-2A, 11A–16A
1994	–	1J–6J, 9J–18J, 16A–21A, 26A–31A
1995	2J–6J	18J–21J, 25A–28A
1996	6A–8A, 16A–22A	7J–12J, 15J–22J, 10A–13A, 23A–29A
1997	9J–13J, 20J–22J, 29A–31A	17J–19J, 23J–26J, 25A–28A
1998	4J–7J, 12J–18J, 3A–5A,12A–15A, 29A–31A	1J–3J, 30J-2A, 20A–22A
1999	10J–13J, 19J–21J, 13A–16A	15J–17J, 24J–27J, 30J-2A
2000	18J–20J, 1A–5A	1J–7J, 28J–30J, 6A–8A, 19A–28A
2001	29J-1A	3J–9J, 16J–19J, 22J–25J, 11A–14A, 18A–20A
2002	1J–5J, 21J–24J, 16A–18A	26J–28J, 31J-2A, 5A–8A
2003	10A–12A	2J–9J, 17J–21J, 23J–27J, 2A–7A
2004	7J–10J	1J–3J, 1A–3A
2005	1–5J, 13J–16J, 6A–11A,21A–25A	7J–10J, 22J–26J, 28J-2A
2006	8J–12J	1J–3J, 13J–16J, 21J–26J, 31J-4A, 11A–14A
2007	1J–3J, 20J–23J, 16A–18A	10J–14J, 2A–6A, 19A–23A,
2008	1J–8J, 12J–16J, 17A–19A	25J-4A, 21A–27A
2009	1J–4J, 28J-1A,6A–9A,19A–23A	13J–15J, 18J–21J, 24J–26J, 2A–4A
2010	11J–13J	1J–10J, 15J–25J, 27J–29J, 2A–9A, 29A–31A
2011	1J–3J, 19J–22J, 5A–11A	5J–7J, 24J–29J, 28A–31A
2012	13J–19J	1J–3J, 20J–23J, 1A–4A,
2013	26J–29J, 17A–19A	2J–12J, 15J–25J, 2A–5A, 12A–14A, 23A–26A
2014	14A–17A	7J–10J, 12J–15J, 24J–27J
2015	8J–10J, 18J–20J, 24J-2A, 17A–21A	12J–14J, 12A–14A
2016	3J–7J, 16J–18J	27J–30J, 2A–5A, 12A–15A, 23A–25A
2017	3J–6J, 20J–22J, 24J–26J, 11A–13A	15J–18J

**Fig. 3:**
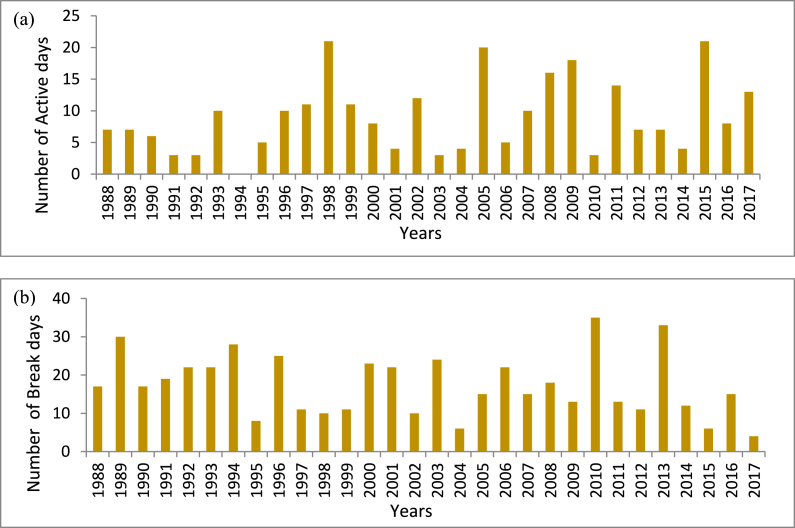
Time series of (a) active days and (b) break days.

Fig. 3 (a) and (b) show the total number of active and break days for a particular year from 1988 to 2017. The highest number of active days (21 days each) are found between 1998 and 2015, while the lowest number of active days (3 days each) was found between 1992, 2003, and 2010. The break spell having the highest number of total 35 break days was found in 2010. The years 1989 and 2013 show 30 and 33 break days respectively. Only 4 break days were found in the year 2017 which is the lowest within 30 years. The numbers of total active days within 30 years are found in the months of July (173 days) and August (98 days). Between 1988 and 2017, there were 306 and 201 break days in the months of July and August, respectively.

Table 2 illustrates that there were no active phases in 1994 and the highest number of active phases (5 phases) was found in 1998. The trend of active phases is found randomly distributed over Bangladesh. It is seen that the active phases usually occur in the month of July. But, there were no active phases in the month of July in 1994, 1996, and 2003. The highest number of active phases (3 phases) were found in 2017 which occurred in the month of July. Most of the years are found to have no active phases throughout the month of August. The year 1998 had 3 active phases in the month of August. Therefore, it is clear that as compared to August, the month of July had more active phases. The highest number of break spells was 6 occurred in 1992 and the lowest number of break spells was 1 occurred in 2017. In most years, the month of July had 2 break phases. Each of the nine years featured three break phases. There are 54 and 43 break phases found in the month of July and August respectively. There was no break phase in 2008 in the month of July. One or two break phases are common in August each year. The years 1988, 2015, and 2017, had no break phases in the month of August. There are 7 break phases found in a combination of July and August. On the other hand, only 2 active phases were found in those months.

**Table 2:** The number of active and break phases in Bangladesh over the period of 1988–2017.

Years	Number of Active phases					Number of Break phases		
	July	August	July+ August	Total	July	August	July + August	Total
1988	1	1	0	2	1	0	1	2
1989	2	0	0	2	2	3	0	5
1990	2	0	0	2	2	2	0	4
1991	1	0	0	1	2	1	0	3
1992	1	0	0	1	3	3	0	6
1993	1	1	0	2	2	2	0	4
1994	0	0	0	0	2	2	0	4
1995	1	0	0	1	1	1	0	2
1996	0	2	0	2	2	2	0	4
1997	2	1	0	3	2	1	0	3
1998	2	3	0	5	1	1	1	3
1999	2	1	0	3	1	1	1	3
2000	1	1	0	2	2	2	0	4
2001	0	0	1	1	3	2	0	5
2002	2	1	0	3	1	1	1	3
2003	0	1	0	1	3	1	0	4
2004	1	0	0	1	1	1	0	2
2005	2	2	0	4	1	1	1	3
2006	1	0	0	1	3	1	1	5
2007	2	1	0	3	1	2	0	3
2008	2	1	0	3	0	1	1	2
2009	1	2	1	4	3	1	0	4
2010	1	0	0	1	3	2	0	5
2011	2	1	0	3	2	1	0	3
2012	1	0	0	1	2	1	0	3
2013	1	1	0	2	2	3	0	5
2014	0	1	0	1	3	0	0	3
2015	2	1	1	4	1	1	0	2
2016	2	0	0	2	1	3	0	4
2017	3	1	0	4	1	0	0	1

From Table 3, the number of active phases having 3 days duration is 27. 16 active phases are having 4 days of lifespan. Almost 67% of the active phases had lifespans of 3–4 days. The active phase is associated with lows and depressions that form over the Bay of Bengal and move across Bangladesh. These synoptic systems often last 3–4 days. There are no active phases of 9 days duration. The longest active spells found were from July 24, 2015 to August 02, 2015 covering 10 days. 28 break phases last 3 days longer and 36 break phases last 4 days longer. Almost 62% of the break phases had lifespans of 3–4 days. Only one break phase had a duration of 12 days from July 16, 1989 to July 27, 1989. There were no active spells that lasted more than ten days. Almost 8.7% of the break spells had more than 10 days of duration. Table 3 clearly shows that the break days having 3–4 days duration are more frequent.

**Table 3:** Variations in the number of days during active and break Phases.

Duration (Days)	Active spells	Break Spells
3	27	28
4	16	36
5	14	10
6	1	9
7	4	6
8	1	4
9	0	1
10	1	4
11	0	4
12	0	1

**Fig. 4:**
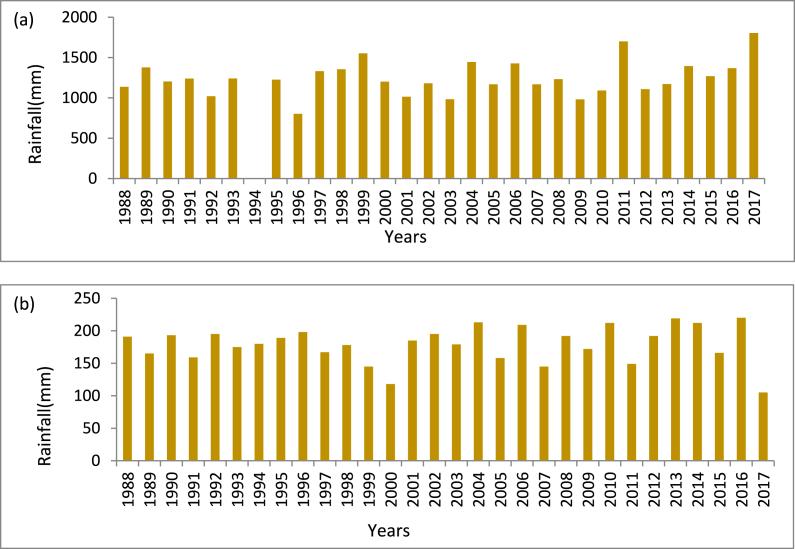
Daily Average Rainfalls of (a) active and (b) break Days over Bangladesh.

Fig. 4 (a) and (b) show the daily average rainfall for a particular year during the active and break phases. Average rainfall per active and break day is found 1207 mm and 179 mm respectively. The highest daily rainfall was found at 1805.23 mm, in the active seasons of 2017. The highest rainfall per break day was 247.09 mm in 2012. The minimum rainfall is 802.6 mm per day in active phases found in 1996. The lowest rainfall per break day was found in 2017 (105.5 mm).

### 3.2 Model simulated active and break monsoon weather patterns over Bangladesh

#### 3.2.1 Rainfall pattern

**Fig. 5:**
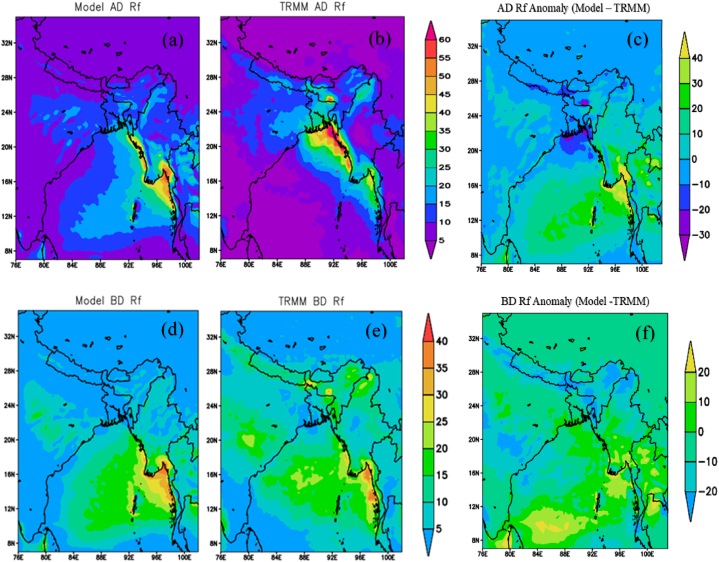
Differences between active (a, b, c) and break (d, e, f) monsoon rainfall between WRF and TRMM. AD, BD, and Rf represent active days, break days, and rainfall respectively.

Fig. 5(a) and (b) depict the 10-year averaged monsoon rainfall per active day, while Fig. 5(c) illustrates the differences between the model and TRMM. The model-simulated value indicates that the highest rainfall (60 mm/day) occurs over the southeastern part of Bangladesh, whereas the observation from TRMM indicates the highest rainfall (65 mm/day) occurring in the same region. Both the model and the observation indicate that most of the rainfall occurs in the northeastern and southeastern regions of the country. The Shillong Plateau and the Chittagong Hill Tracts, which serve as barriers to the southerly or southwesterly monsoon wind, are believed to impact these regions and initiate rainfall. Rainfall in the northeastern region of the country increases when it is situated near the Shillong Plateau. TRMM shows 20–25 mm/day of rainfall in the central region of the country, while WRF shows 10–15 mm/day. TRMM indicates 10–20 mm/day of rainfall in the northern region, whereas WRF displays less than 10 mm/day. In the southern region of Bangladesh, TRMM estimates 40–60 mm of rainfall, while the WRF simulation shows 15–30 mm/day. In the southwestern region of Bangladesh, TRMM estimates 25–40 mm of rainfall, whereas the WRF simulation displays 10–20 mm/day. Fig. 5(c) reveals the highest difference of −30 to −10 mm found over the southern part, and the rest of Bangladesh shows differences ranging from −10 to 10 mm.

The rainfall per break day over Bangladesh is depicted in Fig. 5(d) and (e). Fig. 5(f) illustrates the differences between the model and TRMM. In comparison to an active day, rainfall on a break day was found to be extremely low. The WRF and TRMM estimated rainfall at about 10–15 mm/day and 15–20 mm/day across the eastern region of the county, respectively. TRMM indicates up to 25 mm of rain per day in the northern region, while WRF simulates 5 mm of rain per day. The model-simulated and observed rainfall is around 5 mm/day and 10 mm/day, respectively, for the other parts of the country. Fig. 5(f) displays the highest difference of −20 mm over the northern and eastern parts of Bangladesh, while the difference in the other parts ranges from 0 to 10 mm. Overall, the model underestimates the amount of rainfall throughout the county when compared to the observations from TRMM.

#### 3.2.2 Wind pattern

**Fig. 6:**
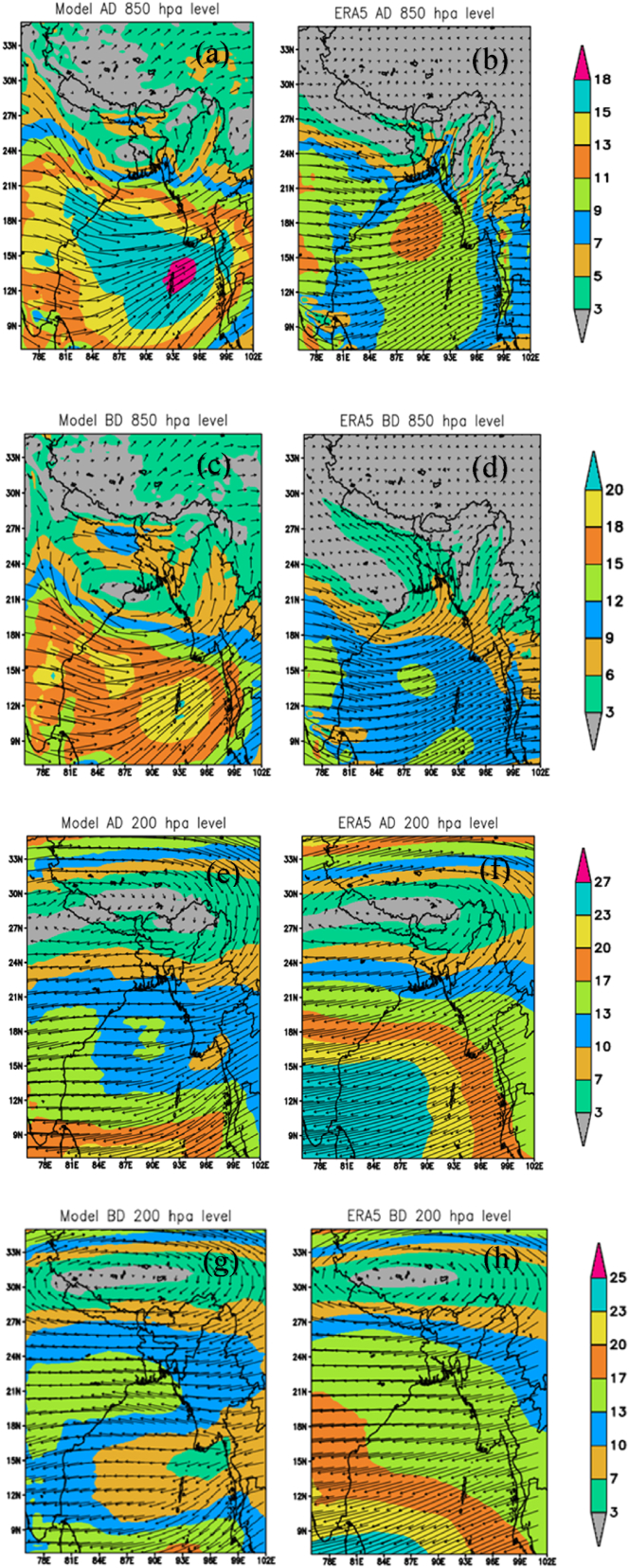
Differences between the model and ERA5 at 850 hPa height winds during active (a, b) and break (c, d) phase of monsoon. And differences between the model and ERA5 at 200 hPa height winds during active (e, f) and break (g, h) phase of monsoon.

Though the area coverage of Bangladesh is small, the upper air circulation of the Southwest summer monsoon has a large impact on the country. Analysing wind patterns is crucial for understanding the monsoon system over an extended period. This study examines wind direction and speed at 850 hPa and 200 hPa during the active and break phases of the monsoon over a 10-year period (2008–2017). Model-simulated wind speed and direction were compared with ERA5 data. fig.6 (a) and 6 (b) show the wind speed and direction per active day (monsoon) over Bangladesh at 850 hPa. WRF shows a cyclonic circulation over the southwestern part of Bangladesh. The monsoon trough can be seen to the north of Bangladesh near the foothills of the Himalayas in both WRF and ERA5. Both WRF and ERA5 indicate that a south-westerly wind is blowing across Bangladesh. The northern and southern parts of Bangladesh have the highest wind speeds, according to WRF, at 5–7 m/s. The south to southeastern portion in parallel is where the wind speed is at its maximum, according to ERA5. WRF shows the lowest wind speed over the central part.

Fig. 6 (c) and 6 (d) show the wind speed and direction per break day (monsoon) at 850 hPa. Both the WRF and ERA5 indicate that the wind comes from the southeast direction. The WRF shows a cyclonic circulation over the extreme southwestern part of Bangladesh. The monsoon trough shifted southward and extended eastward over the region. The WRF shows the wind speed varying from 3 to 9 m/s but ERA5 shows wind speed up to 6 m/s except for the southcentral part of the country. The WRF shows the lowest wind speed is 3 m/s over the southwestern part of the country.

Fig. 6 (e) and 6 (f) show the climatology of wind circulation per active (monsoon) day at 200 hPa. Both the WRF and ERA5 show that the wind flows easterly over Bangladesh on active days. The anticyclonic circulations were found along the foothills of the Himalayan. The wind flows at a speed of 10–13 m/s in the southern region of Bangladesh, according to both WRF and ERA5.

In Fig. 6 (g) and 6 (h), the WRF and ERA5 indicate that the wind flows from the east over Bangladesh on break day at 200 hPa. The anticyclone circulation shifted northward in break days. According to the WRF simulation, the southwestern region of Bangladesh experiences winds with a maximum speed of 13–17 m/s. According to the ERA5 observations, the northern region of Bangladesh experiences winds as low as 10–13 m/s. The rest of the country experiences wind speeds as high as 13–17 m/s.

#### 3.2.3 Sea level pressure

**Fig. 7:**
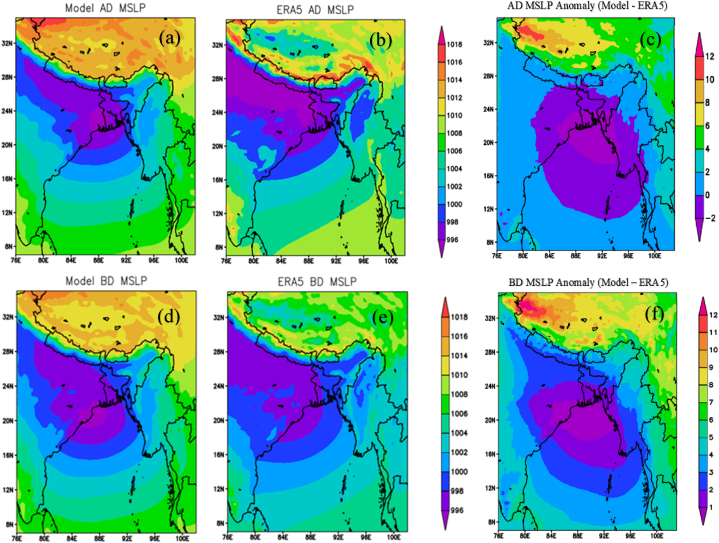
Differences between active (a, b, c) and (b) break (d, e, f) monsoon SLP between WRF and ERA5. AD, BD, and MSLP represent active days, break days, and mean (average) sea level pressure respectively.

Fig. 7(a) and (b), and 7(c) display the SLP patterns during active (monsoon) days. Both the WRF and ERA5 models indicate SLP values around 994–1000 hPa over Bangladesh. The highest SLP values, as shown by both WRF and ERA5, are located over the south-eastern to eastern regions of the country.

In the south-western region, WRF shows SLP values less than 996 hPa, while ERA5 indicates 996 hPa in the western region of the country. Fig. 7(c) illustrates the differences in SLP between the model and observations during active days. The difference ranges from −2 to 0 hPa over Bangladesh.The SLP pattern for each break (monsoon) day is displayed in Fig. 7(d) and (e). Both the WRF and ERA5 models also indicate SLP values of 996–1000 hPa over Bangladesh. WRF exhibits the highest SLP over the southeast and northeast regions. However, ERA5 shows the highest SLP across the entire country except the West. Fig. 7(f) illustrates the differences in SLP between the model and observation during break days. The difference was found to be −1 to 3 hPa over Bangladesh. During active and break monsoons, the values of the lowest and highest SLP ranges were nearly similar, but the spatial distribution exhibited a different pattern. Compared to the break (monsoon) days, most of the areas of the country experience the lowest SLP during active (monsoon) days.

#### 3.2.4 Outgoing longwave radiation

**Fig. 8:**
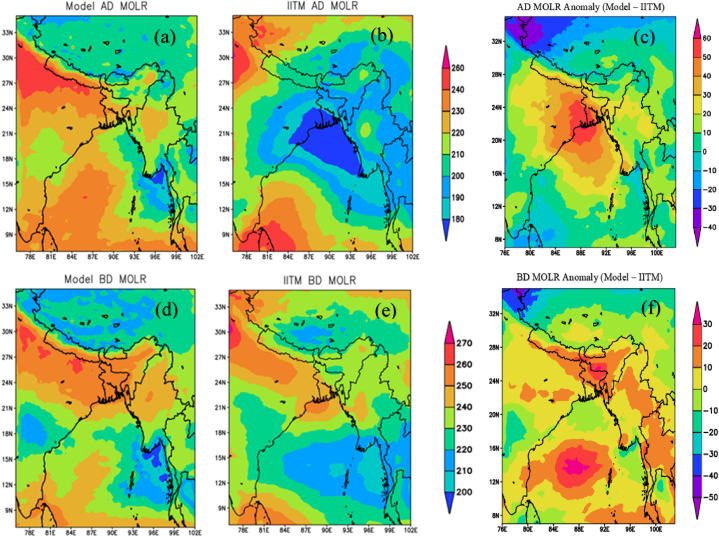
Differences between active (a, b, c) and break (d, e, f) monsoon OLR between WRF and ERA5. AD, BD, and MOLR represent active days, break days, and mean (average) outgoing longwave radiation respectively.

The WRF model was utilized to identify the OLR pattern over a 10-year period (2008–2017) during the active and break phases of the monsoon and to compare it with IITM Kalpana-1 OLR data. Fig. 8(a) and (b) illustrate OLR values during an active day. The WRF depicts OLR values ranging from 210 to 240 W/m^2^, while IITM K-1 OLR data reveals values between 170 and 200 W/m^2^ over Bangladesh. The WRF model displays the lowest value of 210 W/m^2^ over the south-eastern and north-eastern parts, whereas IITM K-1 OLR data indicates the smallest value of less than 180 W/m^2^ over the south-western part of the country. Fig. 8 (c) shows the OLR value difference between the model and observation during an active day. It was found value difference of 20–60 W/m^2^ over Bangladesh. The highest difference was found at 60 W/m^2^ over the southwestern region. Fig. 8 (d) and 8 (e) show OLR during break day, IITM K-1 indicates OLR values of 230–260 W/m^2^ across Bangladesh whereas the WRF reports OLR values of 250–260 W/m^2^. WRF shows a value of 250–260 W/m^2^ all over Bangladesh. The lowest OLR value from IITM K-1 OLR (230–240 W/m^2^), was found in the eastern regions. Fig. 8 (f) shows the OLR value difference between the model and observation during break day. The highest difference was found at 20–30 W/m^2^ over the northeastern part and the lowest difference was found at 0–10 W/m^2^ over the southwestern part. Over Bangladesh, the OLR pattern of an active day is different from that of a break day. Actually, OLR can be considered as an indicator of temperatures at the top of clouds over the tropics including Bangladesh and thus can be used to estimate the intensity of atmospheric convection, which is closely related to rainfall.

## 4 Conclusion

In this study, the rainfall over Bangladesh during the monsoon seasons from 1988 to 2017 has been investigated in terms of the intraseasonal variation of monsoon activities. This approach evaluates the active and break monsoon days over 30 years. Break phase frequencies are higher than active phases of southwest monsoon over Bangladesh. In total, 271 active days and 65 active phases were identified within 30 years. According to this study, 16 active phases and 62 active days were found in the first 10 years (1988–1997), 24 active phases and 98 active days were found in the second decade (1998–2007) and the third decade (2008–2017) also covered 25 active phases and 111 active days. It demonstrates an upward trend in Bangladesh's active days and active phases for monsoon. In total, 39 active monsoon phases were revealed in the month of July, while 23 active phases were detected in the month of August. The remaining three active monsoon spells occurred comprised of July and August. Over the course of 30 years, 104 break phases and 517 break days were identified. This study also identified 37 break phases and 199 break days in the first 10 years (1988–1997), 158 break days and 35 break phases during the second decade (1998–2007), and 32 active phases and 160 break days in the third decade (2008–2017). It shows a declining tendency in the monsoon break phases in Bangladesh. In all, 54 monsoon break phases were detected in the month of July, while 43 break phases were detected in the month of August. Between July and August, the remaining 7 break monsoon spells took place. The active phase is roughly 3–4 days long (67%) and the break phase is 3–4 days long (62%). According to 30 years (1988–2017) of climatology, the average amount of rainfall that occurred during the active and break days is 1207 mm and 179 mm respectively. 10 years (2008–2017) intraseasonal variability of monsoon has been investigated by WRF model simulation and compared with the dataset from TRMM, ERA5, and IITM K-1. By contrasting the characteristics of synoptic-scale monsoon activities between active and break periods identified in the variance of rainfall throughout Bangladesh. When the monsoon trough is at the base of the Himalayas, rainfall increases; when it moves to the south of Bangladesh, it diminishes. The rainfall increases with the south-westerly wind at 850 hPa and decreases with a south-easterly wind at the same level. At 200 hPa level, during active monsoon anticyclone circulation was found in the north of Bangladesh, but it shifted northward during break phases. Active phases consist of low sea level pressure, while break phases have significant sea level pressure. Outgoing longwave radiation was found to be high on break days but low on active days.

## Author contribution statement

Zannatul Ferdoushi: Analyzed and interpreted the data; Wrote the paper.

D. A. Quadir: Conceived and designed the experiments.

S. M. Quamrul Hassan: Performed the experiments; Contributed reagents, materials, analysis tools or data.

## Funding statement

This research did not receive any specific grant from funding agencies in the public, commercial, or not-for-profit sectors.

## Data availability statement

Data will be made available on request.

No additional information is available for this paper.

## Declaration of competing interest

The authors declare that they have no known competing financial interests or personal relationships that could have appeared to influence the work reported in this paper.

## References

[1] Krishnamurthy V., Kinter J.L. (2003). The Indian monsoon and its relation to global climate variability. Global climate: Current research and uncertainties in the climate system.

[2] Singh P., Nakamura K. (2010 Jun 27). Diurnal variation in summer monsoon precipitation during active and break periods over central India and southern Himalayan foothills. J. Geophys. Res. Atmos..

[3] Khan AH. Investigation of Mechanisms to Detect Recurrence of Droughts in South Asia with Special Reference to Pakistan (Doctoral dissertation, COMSATS Institute of Information Technology Islamabad-Pakistan). https://core.ac.uk/download/pdf/90186352.pdf.

[4] Goswami B.N., Lau W.K.M., DE Waliser (2005). Intraseasonal Variability in the Atmosphere–Ocean Climate System.

[5] Ahmed R., Karmakar S. (1993 Nov). Arrival and withdrawal dates of the summer monsoon in Bangladesh. Int. J. Climatol..

[6] Food and Agriculture Organization of the United Nations (2014). https://www.fao.org/3/ca0375en/CA0375EN.pdf.

[7] Ahasan M.N., Chowdhary M.A., Quadir D.A. (2010). Variability and trends of summer monsoon rainfall over Bangladesh. J. Hydrol. Meteorol..

[8] Hossain M.F. (2014). Impact of climate change in Bangladesh: rainfall. International Journal of Agriculture Innovations and Research.

[9] Karmakar S. (2006). Some aspects of monsoon rainfall in relation to monsoon failures in Bangladesh. Journal of Meteorology and Hydrology, (SOHAM-Nepal).

[10] Kripalani R.H., Kulkarni A., Sabade S.S., Revadekar J.V., Patwardhan S.K., Kulkarni J.R. (2004 Aug 10). Intra-seasonal oscillations during monsoon 2002 and 2003. Curr. Sci..

[11] Solomon S. (2007). The physical science basis: Contribution of working Group I to the fourth assessment report of the Intergovernmental Panel on climate change. Intergovernmental Panel on Climate Change (IPCC), Climate change 2007.

[12] Saroha J. Indian Monsoon: Origin and Mechanism. http://ijrar.com/upload_issue/ijrar_issue_409.pdf.

[13] Sikka D.R. (2011). Synoptic and meso-scale weather disturbances over South Asia during the Southwest Summer monsoon season. InThe global monsoon system: Research and forecast.

[14] Walker J., Rowntree P.R. (1977 Jan). The effect of soil moisture on circulation and rainfall in a tropical model. Q. J. R. Meteorol. Soc..

[15] Krishnan R., Zhang C., Sugi M. (2000 May 1). Dynamics of breaks in the Indian summer monsoon. J. Atmos. Sci..

[16] Pai D.S., Sridhar L., Ramesh Kumar M.R. (2016 Jun). Active and break events of Indian summer monsoon during 1901–2014. Clim. Dynam..

[17] Rajeevan M., Gadgil S., Bhate J. (2010 Jun). Active and break spells of the Indian summer monsoon. J. Earth Syst. Sci..

[18] Gadgil S., Joseph P.V. (2003 Dec). On breaks of the Indian monsoon. J. Earth Syst. Sci..

[19] Goswami B.N., Mohan R.A. (2001 Mar 15). Intraseasonal oscillations and interannual variability of the Indian summer monsoon. J. Clim..

[20] Krishnamurthy V., Shukla J. (2007 Jan 1). Intraseasonal and seasonally persisting patterns of Indian monsoon rainfall. J. Clim..

[21] Rajeevan M., Bhate J., Kale J.D., Lal B. (2006 Aug 10). High resolution daily gridded rainfall data for the Indian region: analysis of break and active monsoon spells. Current science.

[22] Ohsawa T., Hayashi T., Mitsuta Y., Matsumoto J. (2000 Dec 27). Intraseasonal variation of monsoon activities associated with the rainfall over Bangladesh during the 1995 summer monsoon season. J. Geophys. Res. Atmos..

[23] Chakraborty A., Krishnamurti T.N. (2008 Aug 15). Improved forecasts of the diurnal cycle in the tropics using multiple global models. Part II: Asian summer monsoon. J. Clim..

[24] Srinivas C.V., Hari Prasad D., Bhaskar Rao D.V., Baskaran R., Venkatraman B. (2015 Sep 11). Simulation of the Indian summer monsoon onset-phase rainfall using a regional model. InAnnales Geophysicae.

[25] Taraphdar S., Mukhopadhyay P., Goswami B.N. (2010 Nov). Predictability of Indian summer monsoon weather during active and break phases using a high resolution regional model. Geophys. Res. Lett..

[26] Srinivas C.V., Hariprasad D., Bhaskar Rao D.V., Anjaneyulu Y., Baskaran R., Venkatraman B. (2013 Apr). Simulation of the Indian summer monsoon regional climate using advanced research WRF model. Int. J. Climatol..

[27] Bhatla R., Ghosh S., Mall R.K., Sinha P., Sarkar A. (2018 Oct). Regional climate model performance in simulating intra-seasonal and interannual variability of Indian summer monsoon. Pure Appl. Geophys..

[28] Raju A., Parekh A., Chowdary J.S., Gnanaseelan C. (2015 Jun). Assessment of the Indian summer monsoon in the WRF regional climate model. Clim. Dynam..

[29] Blandford H.F. (1886). Rainfall of India. Monsoon Monograph, India Meteorological Department.

[30] Bhate J, Unnikrishnan CK, Rajeevan M. Regional Climate Model Simulations of the 2009 Indian Summer Monsoon. 92.60. jf; 92.40. eg. 2012.

[31] Hossain J, Azam AT, Khatun MA, Rafiuddin M. Study of a Heavy Rainfall Event in Bangladesh Using Global Satellite Mapping of Precipitation Data. https://www.researchgate.net/publication/340645373.

[32] Huﬀman G.J., Adler R.F., Bolvin D.T., Gu G., Nelkin E.J., Bowman K.P., Hong Y., Stocker E.F., Wolﬀ D.B. (2007). The TRMM multi-satellite precipitation analysis: quasi-global, multi-year, combined sensor precipitation estimates at ﬁne scales. J. Hydrometeorol..

[33] Muñoz-Sabater J., Dutra E., Agustí-Panareda A., Albergel C., Arduini G., Balsamo G., Boussetta S., Choulga M., Harrigan S., Hersbach H., Martens B. (2021 Sep 7). ERA5-Land: a state-of-the-art global reanalysis dataset for land applications. Earth Syst. Sci. Data.

[34] Mahakur M., Prabhu A., Sharma A.K., Rao V.R., Senroy S., Singh R., Goswami B.N. (2013 Oct 25). A high-resolution outgoing longwave radiation dataset from Kalpana-1 satellite during 2004–2012. Curr. Sci..

[35] Anthes R.A., Kuo Y.H., Hsie E.Y., Low‐ Nam S., Bettge T.W. (1989 Jul). Estimation of skill and uncertainty in regional numerical models. Q. J. R. Meteorol. Soc..

